# Myocardial damage of the entire ventricular region in a patient with acute myocardial infarction

**DOI:** 10.22038/aojnmb.2019.42134.1289

**Published:** 2020

**Authors:** Hirofumi Kawamata, Tatsuya Kawasaki, Hiroki Sugihara, Satoaki Matoba

**Affiliations:** 1 Department of Cardiology, Matsushita Memorial Hospital, Osaka, Japan; 2 Department of Cardiovascular Medicine, Graduate School of Medical Science, Kyoto Prefectural University of Medicine, Kyoto, Japan

**Keywords:** Myocardial infarction, Pyrophosphate, Scintigraphy, Subendocardium, Thallium

## Abstract

Technetium-99m-pyrophosphate (^99m^Tc-PYP) has been used, in combination with thallium-201, to estimate the site and extent of myocardial infarcts. We report a case of acute myocardial infarction with severe coronary disease in which the distribution of ^99m^Tc-PYP was extensive. A 78-year-old man presented with dyspnea, and a diagnosis of non-ST-segment elevation acute myocardial infarction was made. Emergency coronary angiography revealed total occlusion of the proximal portion of the right coronary artery and left circumflex coronary artery with collateral flow from the left anterior descending coronary artery, which also had severe stenoses. Given his comorbidities and preferences, subsequent angioplasty was waived. Dual myocardial scintigraphic imaging, which was performed four days after admission, demonstrated slightly reduced thallium-201 uptake in the inferior wall and apex, whereas ^99m^Tc-PYP was positive in the entire left ventricular subendocardial region and the free wall of the right ventricle. His clinical course was uneventful with conservative treatment and the patient was discharged 20 days after admission in a stable condition.

## Introduction

 Technetium-99m-pyrophosphate (^99m^Tc-PYP) has been used to localize the site and determine the extent of acute myocardial infarction for decades (1, 2). We report a case of acute myocardial infarction in which myocardial scintigraphy with ^99m^Tc-PYP demonstrated diffuse accumulation not only in the left ventricular subendocardial myocardium but also in the right ventricle.

## Case report

 A 78-year-old man was referred to the Department of Cardiology of Matsushita Memorial Hospital for dyspnea. The patient had been stable at a nursing home until approximately 18 hours before presentation, when the staff noticed that his appetite decreased during dinner. The next morning, the patient reported difficulty breathing and exhibited pallor. 

 His medical history included heart failure, cerebral infarction, cognitive impairment, chronic kidney disease, and hypertension. Medications included carvedilol at a dose of 2.5 mg daily, furosemide at a dose of 40 mg daily, isosorbide dinitrate at a dose of 40 mg daily, and laxatives as needed. He quit smoking 10 years earlier after a 48-pack-year history, did not drink or use illicit drugs, and had no known allergies.

 On examination, he was lethargic and drowsy, but responsive to simple commands. His blood pressure was 144/87 mmHg, pulse was 108 beats per minute, body temperature was 36.7 ºC, and respiratory rate was 28 breaths per minute; his oxygen saturation was 79% while breathing ambient air and 94% while receiving oxygen through a face mask with a reservoir bag at a rate of 10 liters per minute. The jugular venous pulse was high at 10 cm of water. Chest auscultation revealed a gallop rhythm and bibasilar lung crackles. The abdomen was soft and nondistended, and there was no edema of the legs.

**Figure 1:**
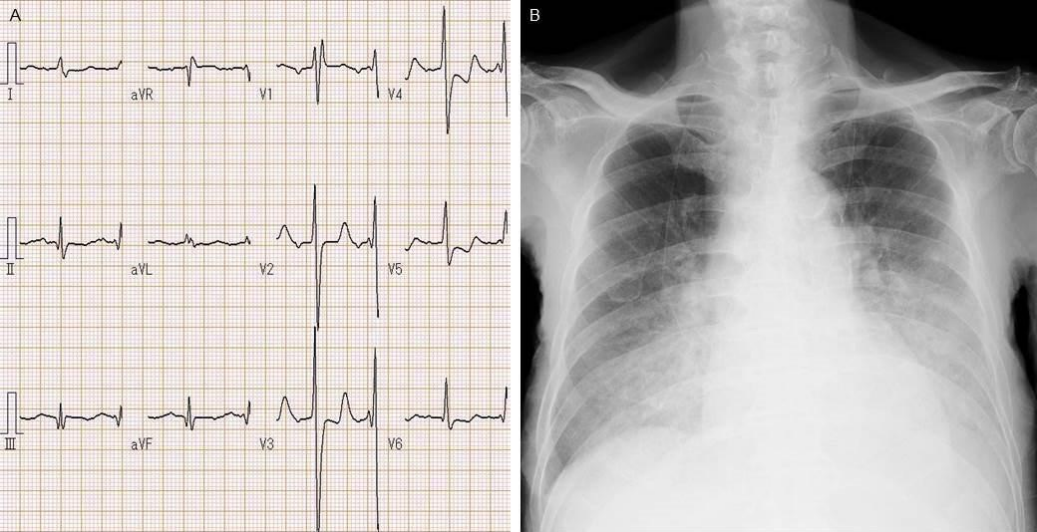
Electrocardiography and chest radiograph

 An electrocardiogram demonstrated a normal sinus rhythm, right bundle branch block, small q waves in the inferior leads, slight ST-elevation in aV_R_, and ST-segment depressions in leads V_3_ to V_6_ (Figure 1A). Bilateral opacities in the lower lung fields were observed on an anteroposterior chest radiograph (Figure 1B), and diffusely decreased wall motion in the left ventricle was noted on bedside echocardiography. Arterial blood gases, obtained with supplemental oxygen, revealed a pH of 7.256, partial pressure of carbon dioxide of 29.5 mmHg, partial pressure of oxygen of 93.8 mmHg, and lactate of 121 mg/dL. His white blood cell count was 13,400 per cubic millimeter with 88.2% neutrophils. The levels of lactate dehydrogenase, total bilirubin, and creatinine were 687 U/L, 2.4 mg/dL, and 2.98 mg/dL, respectively. The levels of creatine kinase and its MB fraction were 1072 U/L and 146 U/L, respectively. The troponin T level was 3.800 ng/mL (reference value, ≤0.100) and brain natriuretic peptide level was 4256.4 pg/mL (reference value, ≤18.4).

The electrocardiogram shows a normal sinus rhythm, right bundle branch block, small q waves in the inferior leads, and ST-segment depressions in leads V_3_ to V_6_; no ST-segment elevation is present, except for in lead aV_R_ (A). Bilateral opacities in the lower lung fields are observed on the anteroposterior chest radiograph (B), findings consistent with lung congestion

**Figure 2:**
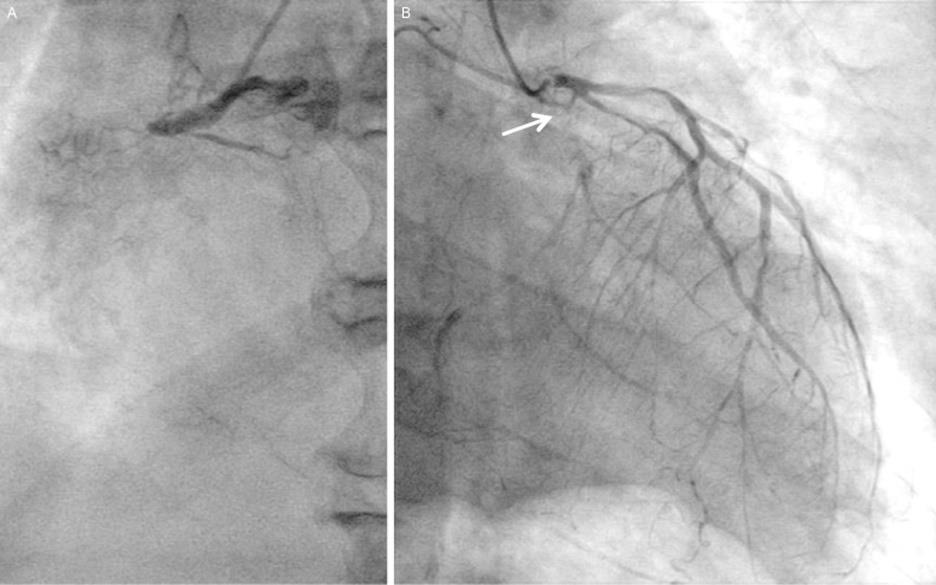
Coronary angiography

 A diagnosis of non-ST-segment elevation acute myocardial infarction accompanying heart failure was made. Emergency coronary angiography demonstrated total occlusion of the proximal portion of the right coronary artery and left circumflex coronary artery with collateral flow from the left anterior descending coronary artery, in which severe stenoses were present at the proximal site and the first branch (Figure 2). Given his comorbidities and preferences, subsequent angioplasty was waived. 

**Figure 3:**
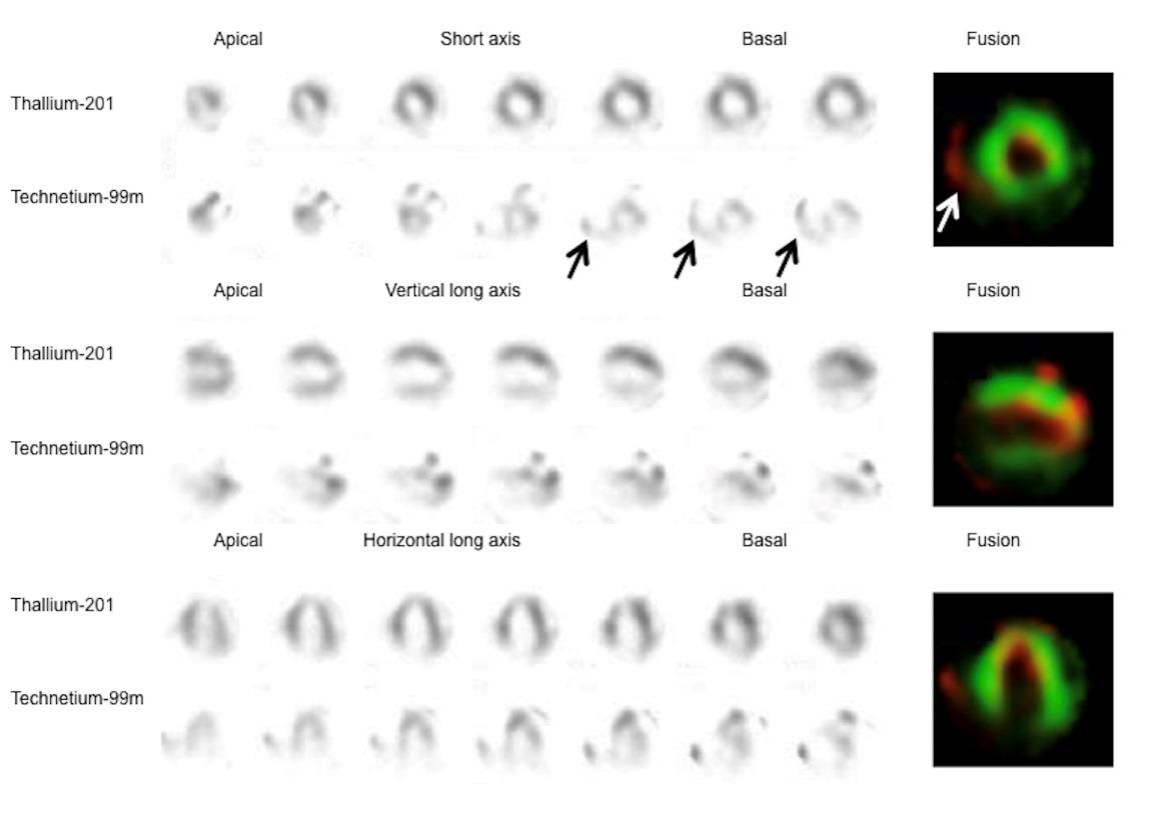
Single-photon emission computed tomography

 Myocardial imaging with thallium-201 (111 MBq) and ^99m^Tc-PYP (740 MBq) was performed four days after admission. A total of 36 images over a 180-degree anterior arc were acquired 5 minutes and 3 hours after tracer injections, respectively, using a digital gamma camera equipped with a low-energy, high-resolution, parallel-hole collimator. The acquisition lasted 50 beats per projection, was stored in a matrix of 64×64 pixels, and the images were reconstructed using a Hanning filter without attenuation or scatter correction. Single-photon emission computed tomography (Figure 3) revealed slightly reduced thallium-201 uptake in the inferior wall and apex, and extensive uptake of ^99m^Tc-PYP in the entire left ventricle and the free wall of the right ventricle. Of note, the uptake of ^99m^Tc-PYP in the left ventricle was distributed to the inner layer with some overlap with that of thallium-201, a finding consistent with myocardial infarction and/or injury in the left ventricular subendocardial regions.

Total occlusion is observed in the proximal portion of the right coronary artery (A) and left circumflex coronary artery (B, arrow). Shown is the collateral flow from the left anterior descending coronary artery mainly to the right coronary artery, and from the intermediate branch and the first diagonal branch mainly to the left circumflex coronary artery. Note the presence of severe stenoses in the proximal site, the first diagonal branch, and intermediate branch of the left anterior descending coronary artery

Myocardial perfusion images with thallium-201 show normal uptake with the exception of a mild reduction in the inferior wall and apex of the left ventricle. Myocardial imaging with ^99m^Tc-PYP demonstrates diffuse tracer uptake not only in the left ventricle but also in the right ventricle (arrows). The fusion images of thallium-201 (green) and ^99m^Tc-PYP (red) show that the uptake of ^99m^Tc-PYP in the left ventricle is distributed to the inner layer of the left ventricle

## Discussion

 The accumulation of ^99m^Tc-PYP, compared with thallium-201 uptake, in the present case was preferentially distributed to the subendocardial myocardium of the left ventricle, indicating subendocardial infarction given the increased cardiac biomarkers. The notable finding was the extent of positive ^99m^Tc-PYP imaging, which included the entire left ventricular subendocardial region and the free wall of the right ventricle.

 Myocardial scintigraphy with ^99m^Tc-PYP is sensitive for the detection of acute myocardial infarcts, regardless of transmural or non-transmural myocardial infarction, if applied during the early phase of acute myocardial infarction (e.g., 24 to 72 hours after the onset of symptoms) (3,4). The exact mechanism responsible for the direct visualization of acute myocardial infarcts remains to be elucidated, but was suggested using a rabbit model that the uptake of pyrophosphate results from the formation of polynuclear complexes with denatured macromolecules rather than from the deposition of calcium in mitochondria (5). Another study (6), however, reported a temporal and topographical relationship between calcium accumulation and pyrophosphate uptake in acute myocardial infarcts in dogs. 

 It may be intuitive that diffuse subendocardial infarction of the left ventricle can be provoked by type 2 myocardial infarction, which is mainly defined as an imbalance between myocardial oxygen supply and demand unrelated to coronary thrombosis (7). The combination of ^99m^Tc-PYP scintigraphy and thallium-201 scintigraphy is effective in detecting subendocardial myocardial infarction in patients without epicardial coronary artery stenosis, e.g., aortic stenosis (8, 9). A similar phenomenon may develop even in patients with type 1 myocardial infarction (i.e., identification of a coronary thrombus by angiography) (7), especially in patients who have severe coronary disease. Possible conditions include obstruction of the left main trunk by a hypoplastic right coronary artery or stenosis of the left anterior descending artery providing collateral flow to an obstructed right coronary artery and left circumflex coronary artery, as observed in our case. Similarly, diffuse uptake of ^99m^Tc-PYP in the left ventricle was demonstrated in a patient with acute subendocardial infarction due to 90% stenosis in the proximal sites of the right coronary artery, left anterior descending artery, and circumflex artery although not single-photon emission computed tomographic images but planar images were used and no accumulation was observed in the right ventricular free wall (10).

 Myocardial scintigraphy with ^99m^Tc-PYP can be used to differentiate necrotic tissue from viable myocardium when compared with the accumulation of thallium-201 or ^99m^Tc -tetrofosmin on myocardial perfusion scintigraphy (11). It is worth noting, however, that ^99m^Tc-PYP may accumulate not only in necrotic myocardium but also in severely damaged but viable myocardium in patients with acute myocardial infarction (12). Given the relatively low value of the peak creatine kinase level in our case, the extent of ^99m^Tc-PYP uptake was likely due to both necrosis and severe damage provoked by type 1 myocardial infarction. Furthermore, increased filling pressure of the left ventricle due to heart failure as a result of type 1 acute myocardial infarction may have caused subendocardial hypoperfusion in areas that were not perfused by the infarct related artery, although the culprit artery has remained undetermined.

 Myocardial scintigraphy with ^99m^Tc-PYP has been used to assess myocardial infarction of the right ventricle (13, 14). The uptake of ^99m^Tc-PYP in the right ventricle was observed in our case, a finding consistent with right ventricular damage, although there were no clinical features suggesting right ventricular infarction such as hypotension, electrocardiographic sign in the right precordial leads, or wall motion abnormalities in the right ventricle. It is well known that ^99m^Tc-PYP scintigraphy is highly specific for detecting right ventricular infarction, although its sensitivity is not excellent (15). The mechanism of right ventricular damage in our case remains unclear, but a possible explanation is right ventricular infarction due to severe coronary disease of the right coronary artery and/or the effects of left-sided heart failure of the myocardium of the right ventricle.

 In conclusion, this case highlights the importance of dual scintigraphic imaging using ^99m^Tc-PYP and thallium-201 for the assessment of myocardial damage, especially when the extent of myocardial damage is undetermined.

## Conflict of Interest

 None declared.
